# Economic Evaluation of anti-epileptic Medicines for Autistic Children with Epilepsy

**DOI:** 10.1007/s10803-023-05941-8

**Published:** 2023-05-04

**Authors:** Michela Tinelli M, Aine Roddy, Martin Knapp, Celso Arango, Maria Andreina Mendez, James Cusack, Declan Murphy, Roberto Canitano, Bethany Oakley, Vinciane Quoidbach

**Affiliations:** 1 Care Policy and Evaluation Centre, London School of Economics and Political Science, London, UK; 2 Department of Child and Adolescent Psychiatry, Institute of Psychiatry and Mental Health, School of Medicine, Hospital General Universitario Gregorio Marañón, Universidad Complutense, CIBERSAM, IiSGM, Madrid, Spain; 3 Autistica, London, UK; 4 Kings College London, London, UK; 5 azienda - Azienda ospedaliero-universitaria Senese, Siena, Italy; 6 European Brain Council, Bruxelles, Belgium; 7 Atlantic Technological University, Sligo, Ireland

**Keywords:** Autism, Epilepsy, Children, Cost-effectiveness, Healthcare expenditure, Family impacts

## Abstract

We examine the cost-effectiveness of treating epilepsy with anti-epileptic medicines in autistic children, looking at impacts on healthcare providers (in England, Ireland, Italy and Spain) and children’s families (in Ireland). We find carbamazepine to be the most cost-effective drug to try first in children with newly diagnosed focal seizures. For England and Spain, oxcarbazepine is the most cost-effective treatment when taken as additional treatment for those children whose response to monotherapy is suboptimal. In Ireland and Italy, gabapentin is the most cost-effective option. Our additional scenario analysis presents the aggregate cost to families with autistic children who are being treated for epilepsy: this cost is considerably higher than healthcare provider expenditure.

The prevalence of epilepsy amongst autistic children is around 7%, rising to 26% in adolescents, in comparison to 1% prevalence in the general population (El Achkar & Spence, 2015; Liu et al., 2022; Zack & Kobau, 2017). Epilepsy has been identified as a leading cause of premature mortality for autistic people (Hirvikoski et al., 2016). Co-occurring epilepsy and autism present greater challenges for clinicians in identifying and treating epilepsy, particularly if someone also has intellectual disabilities (Besag, 2017). Recommended treatments for medical conditions which co-occur with autism (such as epilepsy) are largely similar for both autistic and non-autistic people (National Clinical Guideline Centre, 2012), but the effectiveness and cost-effectiveness of treatment may be different for autistic people compared to the neurotypical population. This is potentially the case for epilepsy, since childhood and adolescent onset are associated with above-average use of long-term healthcare resources, and there may also be long-term negative impacts on education level, employment status, and earned income (Hunter et al., 2015; Jennum et al., 2016; Knapp et al., 2015; Snell et al., 2013).

The National Institute for Health and Care Excellence (NICE) was established in England in 1999 to review available clinical, economic and other evidence to develop guidelines for the (tax-funded) National Health Service (NHS). NICE is a non-departmental public body funded by the government, but independent in its role of developing guidelines. In 2021, NICE updated guidance on the diagnosis and management of epilepsies (NICE, 2021). This guidance applies to the overall population of people living with epilepsy, and not specifically to autistic individuals. The purpose of the study described in this paper is to examine whether recommended treatments have different effects and costs for autistic people with epilepsy. We also aim to examine a wider set of impacts on society (family out-of-pocket expenses, work productivity and informal/unpaid care), since although NICE does not look beyond the health and social care system, autism often has wide-ranging impacts.

We explore the cost-effectiveness of antiepileptic drugs in treating autistic children in four countries with different healthcare systems and practices – England, Ireland, Italy and Spain. An important consideration is that, unlike for a neurotypical population, family impacts (and associated costs) can be sizeable (Buescher et al., 2014). It is therefore important to examine the widest range of costs and effects (if data allow). Our study is part of the [name deleted to maintain the integrity of the review process] project (reference deleted to maintain the integrity of the review process).

## Methods

We first carried out simulation modelling by adapting the approach used by NICE (2021) to estimate the economic impact of anti-epileptic medicines specifically for autistic children with epilepsy. This was done for each of the four study countries from the healthcare system perspective. We then conducted a scenario analysis for the Irish setting (the only country for which suitable data are available) to explore a wider set of impacts: out-of-pocket expenditure, lost earnings and informal (unpaid) care costs.

### Modelling: Impact on Healthcare Providers

The analysis focuses on the cost-effectiveness of antiepileptic drugs (AEDs) for healthcare providers when treating focal seizures (an epileptic seizure starting in one side of the brain). Although no specific type of seizure is associated with autism, focal seizures were chosen as the focus as they are the most common seizure type in children and adults (NICE, 2021). We build on the NICE (2021) model on the cost-effectiveness of pharmacological treatments for a focal seizure and adapt it to reflect the four case study settings. We assume, after consultation with experts, that the NICE model could be applicable to an autistic population of children with focal epilepsy.

#### Model Review

The analysis evaluates the comparative costs and effectiveness of different AEDs used as monotherapy in treatment of focal seizures. The model describes health effects in terms of quality-adjusted life years (QALYs). A QALY measures the state of health of a person or group in which the benefits, in terms of length of life, are adjusted to reflect the quality of life (NICE, 2023). One QALY is equal to 1 year of life in perfect health, compared to death, which is ascribed the value 0 (Whitehead, S. J., & Ali, S. 2010).Costs are described from healthcare providers’ perspective and expressed in 2020 values (in Euros, €). Healthcare provider costs (hospitalization, accident and emergency visits, specialist visits and GP visits) are those associated with starting and switching therapies, additional healthcare support required for the treatment of seizure-free and not seizure-free epilepsy patients and costs of specific anti-epileptic medicines. Given the lack of data for autistic children with epilepsy, we used NICE’s measures of service use and applied these to different country settings. The unit costs were sourced from published sources and expert opinion. Unit costs, health service use, recommended doses, mean 6-month cost for individual AEDs and total costs for starting and switching AED therapy are provided in Appendices 1 and 2.

Treatment effectiveness is reported as: response or non-response (proportions who are seizure-free or not) to monotherapy as well as adjunctive therapy (where we have typically monotherapy first and the addition of adjunctive treatments in those whose response is suboptimal), withdrawal (due to adverse events; AEs) and -QALY gains (often called ‘utility’ in health economic evaluations). The performance of different treatment sequences is estimated using a cost-consequence approach in alignment. We calculated incremental cost-effectiveness ratios (ICER) as used in NICE appraisals: these measure the difference in cost between two alternative treatments divided by the difference in QALY gains.

Costs and utilities for different treatment strategies are calculated over a 15-year time horizon for a hypothetical cohort of 6-year-old children with newly diagnosed focal seizures. The value of costs and benefits were adjusted for the time they occurred using a health economic technique called discounting (Drummond et al., 2005). According to normal practice, we applied a rate of 3.5%.

#### Comparators

Based on the indications listed in the British National Formulary (BNF), drugs licensed as monotherapy for focal seizures in children are carbamazepine, lamotrigine, levetiracetam, oxcarbazepine, sodium valproate and topiramate. Oxcarbazepine and topiramate are only licensed as monotherapy in children over 6 years of age, lamotrigine in children over 12 years and levetiracetam in children over 16 years. Gabapentin and oxcarbazepine are licensed for children over 6 years and levetiracetam and tiagabine are only licensed as adjunctive therapy for children aged over 12. The specific drugs included in the evaluation of monotherapy AEDs are determined by the availability of clinical trial data on outcomes (efficacy and tolerability). On that basis, only carbamazepine, lamotrigine and oxcarbazepine are included in the evaluation for monotherapy; and gabapentin, lamotrigine, levetiracetam, oxcarbazepine and topiramate are included in the evaluation of adjunctive therapy.

#### Model Structure

**Fig. 1:**
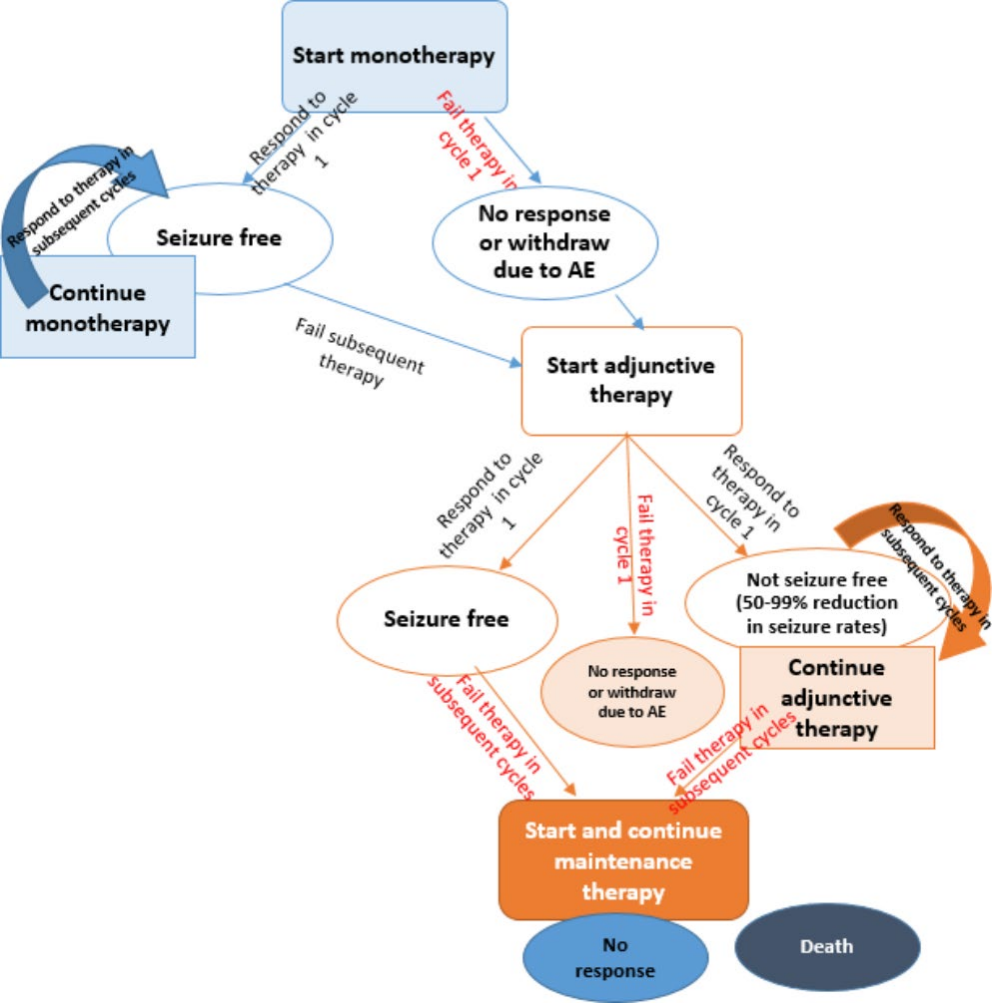
Impact on healthcare providers: Decision-analytic model of treatment with different antiepileptic medicines. (adapted from NICE, 2021) Note AE = adverse event.

The structure of our decision-analytic model is adapted from that developed by Hawkins et al. (2005). It assumes that all hypothetical patients entering the model are newly diagnosed, treatment-naive children with focal seizures. All hypothetical patients start monotherapy and experience one of three outcomes: achieve seizure-freedom; do not achieve seizure-freedom (no response); or withdraw due to adverse events. The model includes two separate states or conditions. Patients who achieve seizure-freedom are assumed to continue the drug treatment for subsequent cycles. Patients who do not achieve seizure-freedom (non-responders and those withdrawing due to adverse events) are assumed to move on to adjunctive therapy to address uncontrolled seizures. The model and how patients move through the pathway is illustrated in Fig. 1.

#### Mortality

Children who achieve seizure-freedom with treatment face the age-dependent death rate associated with experiencing no seizures. Children who experience a reduction or no response to treatment face age-dependent death rates associated with experiencing seizures. These rates and how they were derived are detailed elsewhere (NICE, 2021).

#### Utilities

The QALYs gained for patients in each health state are calculated based on utility estimates associated with being seizure-free, having a 50–99% reduction in seizure frequency or not responding to therapy (i.e., continuing to have uncontrolled seizures). The utility weights used in the model are based on responses from paediatric neurology experts and the literature (Frew et al., 2007). Details are reported elsewhere (NICE, 2021).

#### Sensitivity Analysis

We perform several sensitivity analyses to test the robustness of our cost-effectiveness model (Andronis et al., 2009). In one-way deterministic sensitivity analyses, we vary unit costs and QALY estimates in the model by given amounts and examine the impact on the model results. We also perform probabilistic sensitivity analysis by randomly varying data inputs in our model simultaneously. We therefore examine the effect of joint uncertainty in the variables of the model. We visually inspect the confidence ellipse on the cost-effectiveness plane to identify the region containing 95% of the uncertainty. We also determine the probability that a particular treatment modality is cost-effective compared with placebo, using cost-effectiveness acceptability curves.

### Modelling: Impacts on Families

#### Data

Due to a lack of data for other countries, our analysis of the impacts on families is limited to Ireland. Irish data used to calculate the economic impact of autism and epilepsy on families was previously collected as part of a mixed-methods study on the economic impact and unmet needs of autistic children in 2015 (reference deleted to maintain the integrity of the review process).

#### Estimation of Costs

Irish unit costs for service utilization are based on Health Services Executive (HSE) (2021) data. If Irish unit costs are not available, UK unit costs are applied (Curtis & Burns, 2015). Medication costs are priced using MIMS Ireland (2019). Out-of-pocket expenditure, lost earnings and informal care costs per child based on data collected in Ireland in 2015 are adjusted to 2020 costs (reference deleted to maintain the integrity of the review process).

#### Analysis

Estimates of the association between autistic children with epilepsy on medication and out-of-pocket expenditure are obtained by implementing generalized linear models (GLMs). Out-of-pocket expenditure, lost earnings and informal care costs per child are calculated for all autistic children with epilepsy on medication and autistic children without epilepsy for two age categories: 6 to 10 years and 11 to 13 years. Scenario analyses for the economic impact for families with an autistic child on epilepsy medication compared to an autistic child without epilepsy are based on 7-year, 10-year and 15-year timeframes. This entails using annual cost data for children aged 6–10 years up to year 4 of the model. Cost data for year 5 onwards are based on children aged 11–13 years. We again apply a 3.5% discount rate to costs beyond the base year. No family cost data are available for England, Italy or Spain, and so total family cost for Ireland over 15 years (scenario 3) is used to calculate a ratio of total family costs to healthcare providers’ costs for all four countries.

#### Sensitivity Analysis

Two separate sets of sensitivity analyses are conducted for the family impact costs. First, home adaptations are added to the GLM estimates for out-of-pocket expenditure, resulting in new estimates for total family impacts costs and scenario analysis for 7-year, 10-year and 15-year timeframes. Second, a 4% discount rate is applied to the original results following guidelines for economic evaluations in Ireland (HIQA, 2020) to recalculate the scenario analysis for 7-year, 10-year and 15-year timeframes instead of the 3.5% discount rate recommended in the UK (as used in the main analysis).

## Results

### Modelling: Impact on the Health Care Provider

#### Base Case Analysis

As an example, for England, the total cost per child is €1,990 when starting AED therapy and €2,455 when switching to AED therapy. More information on health service use and total costs for starting and switching AED therapy (for the four nations) is reported in Appendix 3.

The breakdown of costs shows that differences in the cost of individual AEDs are likely to be a determining factor in the total costs of each treatment option (Appendix 4). For example, in England, based on the breakdown of costs, the largest single component of total costs for monotherapy AEDs across the different treatment strategies is the cost of outpatient attendances (61–66%), followed by medicines (21–25%), inpatient costs (9–10%) and other primary care/A&E costs (about 2%). If we consider the costs for adjunctive AEDs, the largest single component of total costs (across the different treatment strategies) is the cost of outpatient attendances (69–73%), followed by medicines (18–25%), A&E costs (6–7%), and other healthcare costs (about 2%).

Analyses for England, Ireland, Italy and Spain demonstrate the effectiveness and cost-effectiveness of carbamazepine as the optimal medicine to try first in children with newly diagnosed focal seizures (Table 1; monotherapy). Summary results per child receiving adjunctive therapy AEDs are presented in Table 2 and vary by country. For England, continued monotherapy (comparator for our economic model) has the lowest total cost and worst outcomes compared with any adjunctive therapy. Levetiracetam generates the greatest gain in QALYs; the ICER is just €23,452, which is just above the willingness-to-pay threshold of £20,000 per QALY which NICE uses to guide whether it will recommend a treatment for use across the NHS. Oxcarbazepine is potentially the most cost-effective adjunctive AED with an ICER of €15,377 (for Spain it is both cost-effective and cost-saving; ICER is - €20,732). By contrast, in Ireland and Italy, gabapentin is the most cost-effective option.

**Table 1:** Impact on healthcare providers: summary results per child receiving monotherapy (15 years)

		England		Ireland		Italy		Spain	
	Total QALYs	Total Costs (€; 2020)		Total Costs (€; 2020)		Total Costs (€; 2020)		Total Costs (€; 2020)	
Carbamazepine	10.18	27,627	Preferred*	9,658	Preferred*	6,690	Preferred*	7,377	Preferred*
**Lamotrigine**	10.09	27,686	Dominated	14,552	Dominated	12,385	Dominated	11,013	Dominated
**Oxcarbazepine**	10.02	30,694	Dominated	12,750	Dominated	8,045	Dominated	9,358	Dominated

*More effective and cost-saving than other alternatives

**Table 2:** Impact on healthcare providers: summary results per child receiving adjunctive therapy (15 years)

		England		Ireland		Italy		Spain	
	Total QALYs	Total Costs (€; 2020)	ICER (€; 2020)	Total Costs (€; 2020)	ICER (€; 2020)	Total Costs (€; 2020)	ICER (€; 2020)	Total Costs (€; 2020)	ICER (€; 2020)
**(Continued monotherapy)$**	9.41	23,495	(comparator)	9,647	(comparator)	8,057	(comparator)	10,208	-
**Gabapentin**	9.46	**24,100**	**12,083^**	**7,128**	Preferred*	**5,381**	Preferred*	9,599	Dominated
**Lamotrigine**	9.47	24,290	Dominated	13,694	Dominated	10,692	Dominated	9,944	Dominated
**Topiramate**	9.51	25,376	Dominated	12,838	Dominated	7,757	Dominated	8,456	Dominated
**Levetiracetam**	9.52	26,169	Dominated	8,426	Dominated	10,496	Dominated	7,886	Dominated
**Oxcarbazepine**	9.52	**25,218**	**15,657^**	8,806	Dominated	6,084	Dominated	**6,721**	Preferred*

$ comparator; ^ More cost-effective than comparator; *More effective and cost-saving than comparator

ICER (incremental cost effectiveness ratio) = cost per additional QALY gained

#### Sensitivity Analysis

In one-way deterministic sensitivity analyses, we evaluate the impact of uncertainty on cost and outcome data. Based on the information available, one-way sensitivity analyses indicate that carbamazepine is the preferred option for monotherapy across all case studies. For example, in England deterministic sensitivity analysis for adjunctive therapy confirms that gabapentin, the lowest cost and least effective AED, is very cost-effective compared to continued monotherapy; and oxcarbazepine is potentially the most cost-effective option across scenarios (apart from when increasing costs or decreasing QALYs by 25% or more, which is a substantial adjustment to assume in these analyses).

Probabilistic sensitivity analyses yield results consistent with the base-case analyses. For example, in England, oxcarbazepine has a 100% probability of being cost-effective (regardless of willingness-to-pay thresholds) to gain an additional QALY (compared with monotherapy).

### Modelling: Impacts on Families

#### Base Case Analysis

Table 3 presents differences in total Irish annual family costs per child: €30,437 for autistic children with epilepsy on medication and €20,867 for autistic children without epilepsy. The breakdown of total annual family costs for the two age groups is as it follows: €37,724 vs. €21,149 (age group 6 to 10 years) and €33,796 vs. €16,966 (age group: 11 to 13 years). The Irish sample estimates for the age 6 to 13 years age category are based on a sample of six children who are autistic, have epilepsy and were on epilepsy medication. Five of the six children were male. There were eight autistic children with epilepsy within the Irish sample, but two children were excluded from the analysis due to not being on medication and not falling within the 6–13 years age category.

**Table 3:** Discounted total family costs per child in Ireland across three scenarios for children aged 6 years onwards

Ireland		
	Autistic child with epilepsy on medication (€; 2020)	Autistic child without epilepsy (€; 2020)
**Total annual family cost per child (6 to 13 years)**	30,437	20,867
**Total annual family cost per child (6 to 10 years)**	37,724	21,149
**Total annual family cost per child (11 to 13 years)**	33,796	16,966
**Scenario 1** **7-year time frame**	258,800	140,254
**Scenario 2** **10-year time frame**	333,220	177,613
**Scenario 3** **15-year time frame**	441,505	231,917

Results of scenario costs between the two groups in Ireland over three timeframes were: scenario 1 (7-year time frame) €258,800 vs. €140,254, scenario 2 (10-year time frame) €333,220 vs. €177,613 and scenario 3 (15-year time frame) €441,505 vs. €231,917. The ratio of Irish long-term family costs to healthcare providers’ costs in England, Ireland, Italy and Spain is presented in Table 4. Due to the lack of family costs data for the other countries, a ratio of long-term family costs based on scenario 3 for Ireland to healthcare providers’ costs in all the case study countries is employed. Italy has the highest difference, with family costs being 66 times more than monotherapy with carbamazepine and 82 times more than adjunctive therapy-gabapentin.

**Table 4:** **Ratio of long-term family costs to healthcare providers costs for England, Ireland, Italy and Spain**

Ratio of long-term family costs to healthcare providers costs (€; 2020)		
**England**	Almost 16 times more than **monotherapy** with carbamazepine (27,627)	18 times more than **adjunctive therapy** - Gabapentin (24,100) Almost 18 times more than **adjunctive therapy** -Oxcarbazepine (25,218) (potentially the most cost-effective)
**Ireland**	Almost 46 times more than **monotherapy** with carbamazepine (9,658)	Almost 62 times more than **adjunctive therapy** - Gabapentin (7,128)
**Italy**	Almost 66 times more than **monotherapy** with carbamazepine (6,690)	82 times more than **adjunctive therapy** - Gabapentin (5,381)
**Spain**	Almost 60 times more than **monotherapy** with carbamazepine (7,377)	Almost 66 times more than **adjunctive therapy** -Oxcarbazepine (6,721)

Note: Ratio of long-term family costs to healthcare providers costs based on scenario 3 (15-year-time frame =€441,505).

#### Sensitivity Analysis

Two separate sets of sensitivity analyses are conducted for the family impact costs and ratios presented in Appendix 5. Including home adaptations in the out-of-pocket dependent variable for GLM estimates results in an increase in overall total family costs from €30,437 to €36,839 for autistic children on epilepsy medication. Scenario 3 total family costs increase from €441,505 to €564,561 resulting in higher ratio results. Changing the discount rate from 3.5% (as used in the UK) to 4% (following Irish guidelines) results in lower ratio results in comparison to the original results.

## Discussion

Our study provides evidence of the cost-effectiveness of antiepileptic drugs in treating autistic children in four countries with different healthcare systems and practices (England, Ireland, Italy and Spain). A clear finding, based on the effectiveness and cost-effectiveness calculations, is that carbamazepine is the optimal drug to try first in children with newly diagnosed focal seizures. For England and Spain, oxcarbazepine is potentially the most cost-effective *adjunctive* AED. In Ireland and Italy, gabapentin is the preferred option. Our analysis highlights the substantial economic impact on families with an autistic child who has epilepsy, which is considerably higher than healthcare expenditure.

Our study has both strengths and weaknesses. The cost-effectiveness analysis was based on a robust model developed by NICE for England and adapted, with the support of experts, to measure the impact on healthcare providers across different country settings. Due to the paucity of data available for autistic children with epilepsy, we assumed that the economic model of pharmacological treatments for children with focal epilepsy used by NICE would apply to autistic children with focal epilepsy. The modelling describes the treatment pathway for focal seizures. Although focal types are the most common in children with epilepsy, this may limit the model’s relevance for autistic children with other types of epilepsy. Also, the parameters for the healthcare model rely heavily on the NICE model. Demographic characteristics such as family income, parental education, comorbidity, or degree of autism are possible influences on QALYs, but the NICE model is based on randomised controlled trials and the methodology of randomisation would have wiped out their effects. Importantly, this is the first study to estimate the extra economic impact on families where there is an autistic child on epilepsy medication in comparison to autistic children without epilepsy. Our findings highlight the extra costs and challenges (related to out-of-pocket expenditure, lost earnings and informal care costs) faced by these families in comparison to the neurotypical population. (The NICE model did not look at family costs when considering the cost-effectiveness of anti-epileptic medication.) It should be noted, however, that the only relevant data set available to us included only seven autistic children with epilepsy on medication. There is only one female within this subgroup in the sample, therefore it was not possible to do an analysis based on gender and we acknowledge this is a limitation of the paper. While Samba Reddy (2017) acknowledges there are gender differences in the susceptibility to seizures, Perucca et al. (2014) found that there is not sufficient evidence to support considerable changes in the efficacy of AEDs due to gender. In relation to potential sample bias, the Irish survey cost data may not be representative of all families with an autistic child in Ireland. However, we are unable to determine whether this leads to any bias in the results.

Previous literature on the cost-effectiveness of epilepsy medication has focused entirely on the neurotypical adult population (Allers et al., 2015; Wijnen et al., 2017; NICE, 2021). Even then, the number of economic evaluations focused on children is very limited (Lee et al., 2013, 2014; Widjaja et al., 2021; NICE, 2021) and none of them looked at implications for autistic people. Healthcare costs vary along the care pathway for epilepsy and are mainly driven by hospitalization costs, followed by specialist (outpatient) visits and medications (Widjaja et al., 2021). Following the literature, hospitalisations accounted for the largest proportion of costs for pre-diagnosis, initial intervention and final care. For ongoing care (described in our model), previous studies and our own analyses show that costs related to specialist (outpatient) visits are one of the main cost-drivers as well as medication costs. Remote consultations with healthcare professionals could secure efficiency gains for the healthcare provider by reducing waiting times and unnecessary appointments for patients who are seeking access to specialist outpatient services (Almathami et al., 2020). They could also secure better outcomes for children, avoid the stress of travel and the busy and noisy environment of a clinic as children could have the consultation in their own safe space (Autistica, 2022). This would bring additional economic savings for their families, cutting down time spent travelling and away from their employment.

The wider literature shows that families with autistic children often experience considerable extra costs, lost earnings and increased caring demands due to their child’s additional needs (Barrett et al., 2012; Buescher et al., 2014; Dillenburger et al., 2015; Hussain et al., 2020; Järbrink et al., 2000; additional reference deleted to maintain the integrity of the review process). A national survey conducted in Ireland found that the average annual cost per autistic child for families was €28,465 because of out-of-pocket expenditure on private autism services, lost income and informal care (reference deleted to maintain the integrity of the review process).

Age is a major influence on family costs for children with epilepsy – costs are higher for older children – but less so for those with no epilepsy (Argumosa et al., 2004). Epilepsy is a chronic neurological disorder that not only has economic impacts but also may have major effects on individual social competence and family relationships (Jennum et al., 2016). This may be even more pronounced in older children and young adults (compared with younger children) for whom the disorder influences self-perception (Hirfanoglu et al., 2009), stigma (Jacoby et al., 2007), education (Fleming et al., 2019) employment (Jennum et al., 2011), social prognosis, and income. Also, there is evidence that children with epilepsy have higher welfare costs compared with people without epilepsy (regardless of comorbid disorders; Jennum et al., 2016).

Our findings have implications for policy and practice. Treating autistic children with epilepsy with medication is cost-effective for the healthcare provider. Carbamazepine, which is widely recommended as a first-line antiepileptic drug for new-onset partial seizures with or without generalization (NICE, 2021), was the preferred monotherapy option for all countries. It presented the lowest proportion of medicine costs (varying between 2% and 20%) across countries. However, different strategies may be needed in different countries for adjunctive therapy. The preferred options (oxcarbazepine for England and Spain and gabapentin for Ireland and Italy, respectively) presented a lower proportion of medicines costs (between 16% and 22%) compared with other options (where the costs of the medicines reached up to 59% of the total costs, as reported by lamotrigine and oxcarbazepine for Italy).

In Ireland, long-term family costs for autistic children treated for their focal epilepsy (€441,505 for 15 years) are likely to be 46 times more than costs borne by healthcare providers: either €9,658 for children on monotherapy with carbamazepine or €7,128 for those on adjunctive therapy with gabapentin. From a family perspective, it is important to understand the lived experience and complexity of the needs involved in caring for an autistic child or adolescent with epilepsy, linked to the unpredictability and frequency of seizures. These needs and uncertainties create extra caring demands which may directly affect the ability of parents to sustain employment if they need to take time off to support and monitor their child and accompany them to medical appointments. We also found that families with autistic children on epilepsy medication had high out-of-pocket expenditures perhaps because of hospital visits or payment for privately paid neurologists and other appointments (due to long waiting lists). Taking family costs into account when considering the economic case for treatment can sometimes alter the recommended decision (Lavelle et al., 2019).
